# Assessing Dental Service Quality Using the SERVQUAL Model: A Cross‐Sectional Study Among Perspectives of Students, Professors, and Nurses at Kerman School of Dentistry

**DOI:** 10.1002/hsr2.72019

**Published:** 2026-03-26

**Authors:** Fatemeh Sadat Sajadi, Zahra Salari, Keyhaneh Ebrahimi, Maryam Rad, Hossein Akbari‐Hooye, Milad Mollaali

**Affiliations:** ^1^ Department of Pediatric Dentistry, Social Determinants on Oral Health Research Centre Kerman University of Medical Sciences Kerman Iran; ^2^ Department of Pediatric Dentistry Kerman University of Medical Sciences Kerman Iran; ^3^ School of Dentistry Kerman University of Medical Sciences Kerman Iran; ^4^ Oral and Dental Diseases Research Center Kerman University of Medical Sciences Kerman Iran; ^5^ Department of Psychology, Faculty of Psychology and Educational Sciences University of Sistan and Baluchestan Zahedan Iran; ^6^ Department of Biology, Faculty of Science University of Sistan and Baluchestan Zahedan Iran

**Keywords:** dental education, dental faculty, dental service quality, Iran, SERVQUAL model

## Abstract

**Background and Aim:**

The quality of dental service provision has a special place in dentistry. This study investigated the quality of dental service provision based on the SERVQUAL model from the perspective of professors, students, and nurses at the School of Dentistry of Kerman University of Medical Sciences in 2021–2022.

**Methods:**

A cross‐sectional descriptive‐analytical study was conducted among 410 participants, including professors, students, and nurses. Data were collected using the SERVQUAL questionnaire assessing six dimensions of service quality: tangibility, reliability, responsiveness, assurance, empathy, and accessibility.

**Results:**

The data revealed that there was a significant difference in the perception of professors and students about the quality of dental services in tangibility, reliability, responsiveness, and accessibility, while empathy and assurance were rated similarly. A significant difference was observed between the perceptions of students and nurses about tangibility and responsiveness.

**Conclusion:**

The professors, students, and nurses in this study had moderately positive attitudes toward the quality of dental service delivery at the Kerman School of Dentistry. The highest score was assigned to service assurance, and the lowest score was assigned to service accessibility.

## 1 Introduction

The development of healthcare organizations in today's competitive environment requires continuous improvement in the quality of healthcare services and the adoption of patient‐centered strategies. Identifying and monitoring factors that influence service quality (SERVQUAL) is essential for achieving institutional goals such as enhancing clinical performance, gaining credibility, and increasing patient satisfaction. Healthcare managers play a critical role in implementing quality improvement initiatives and ensuring that patient expectations are met. In modern management, quality is defined by customer demand, and SERVQUAL is considered a key determinant of organizational success. This is particularly true in healthcare, where SERVQUAL directly affects human life and well‐being. Despite growing attention to patient satisfaction and service standards in healthcare institutions, limited research has focused on evaluating satisfaction and SERVQUAL in dental settings [1, 2, 3, 4].

Given that dental schools play an important role in promoting oral and dental care, students should strive to find a balance between patient needs and student needs by focusing on the fact that “patients and their satisfaction are paramount to student education.” Accordingly, both service providers and patients benefit from their interaction, and patient satisfaction receives priority. In other words, the educational and clinical experiences that dental care providers and academics obtain should be patient‐centered [5]. Pritchett et al. defined satisfaction as “a person's feelings of pleasure or disappointment resulting from comparing a product's perceived performance (or outcome) in relation to his or her expectations” [6].

Measuring the quality of services from the viewpoint of clients and patient satisfaction is considered one of the internal measures that shows the orientation of organizations toward quality improvement. Quality means to provide the best product or service in the best possible way according to the expectations of the customer (patient). Thus, taking into account the expectations and achieving them is one of the important concepts in the success of dental centers [7].

The SERVQUAL instrument traditionally includes five dimensions: tangibility, reliability, responsiveness, assurance, and empathy [8]. However, in healthcare and dental service contexts, accessibility, including ease of appointment, physical and organizational access, has been identified as a critical aspect of SERVQUAL; recent studies in health and dental services [9] have extended SERVQUAL to include accessibility as a sixth dimension. The SERVQUAL model thus defines SERVQUAL as a multidimensional construct comprising these five validated domains, tangibility, reliability, responsiveness, assurance, and empathy, which collectively reflect customers' expectations and perceptions [10], and, in health and dental care contexts, recent research has expanded this framework by adding accessibility, encompassing factors like ease of access, appointment availability, and organizational infrastructure, as a sixth dimension [11]. Accordingly, this study adopts all six dimensions to capture both interpersonal and structural components of dental SERVQUAL and to align our measurement with contemporary SERVQUAL applications in health services.

Dental centers in Iran are among the medical centers that provide unique services, and their success requires the high expertise of dentists and specialized materials and equipment. Dental centers should have the necessary dynamics in this regard. One of the missions of universities in training dentists is to provide education oriented to patient satisfaction. Hence, it is necessary to develop and put into practice curricula that promote students' knowledge and skills. In addition, the degree of patient satisfaction reflects the efficiency of the teaching methods [4].

The existence of competition among dental centers due to the cost of services and efforts to obtain more patient satisfaction and a greater share of patient referrals and treatment requests has caused medical centers to seek a privileged position in the market. Besides, patients search for clues to reach the best dental centers [8].

John et al. found that the most important aspects of SERVQUAL were responsiveness, empathy, and reliability of dental and healthcare service providers. There was a considerable difference between patients' expectations and their perceptions of the dimensions of SERVQUAL [12]. Pekkaya et al. stated that the quality of medical services was considerably different in terms of demographic factors of patients, such as age, income, and type of services. However, the quality of medical services did not show any significant difference in terms of gender, marital status, education, or occupation. Additionally, reliability was observed as the most determining dimension for the satisfaction of outpatients [13]. Anisseh et al. found that patient satisfaction with dental care was positively correlated with good SERVQUAL [14]. Dopeykar et al. revealed that the amount and quality of services that are provided in a dental clinic do not meet the expectations of patients and service receivers. Moreover, the maximum and minimum quality gaps were associated with the dimensions of empathy (16.1) and reliability (0.61), respectively [15].

In recent years, the SERVQUAL model has been widely used in Iran and other countries to assess healthcare and dental SERVQUAL, often highlighting deficiencies in responsiveness, empathy, and reliability. However, most studies have focused on hospitals or private clinics, with limited attention to academic dental institutions. Despite growing interest in SERVQUAL, little is known about how various stakeholders, such as professors, students, and nurses, perceive SERVQUAL in educational dental settings. At the Kerman School of Dentistry, significant structural and educational reforms over the past decade, including curriculum updates, increased clinical capacity, and expanded staffing, underscore the need for a current SERVQUAL‐based evaluation to inform quality improvement and planning. Although expectations and their fulfillment are key to dental center success, only one qualitative study (2009) has explored SERVQUAL at this institution through interviews with patients, staff, and students [16]. Consequently, there is no reliable, up‐to‐date evidence available to guide oversight and decision‐making. Given the substantial developments since 2009, such as the admission of residents and assistants, physical expansion, increased student intake, and curriculum revision, an updated assessment is essential. Moreover, aligning patient satisfaction with student training is critical to fostering constructive provider–recipient interactions and enhancing educational outcomes.

Therefore, the present study aimed to evaluate the quality of dental service provision using the SERVQUAL model and to compare perceptions of SERVQUAL among professors, students, and nurses at the Kerman School of Dentistry, Kerman University of Medical Sciences, during the 2021–2022 academic year. A comparison of the results from the present study and previous studies in the literature can contribute to identifying and resolving possible research gaps. The insights from this study can also help officials, managers, and heads of departments, colleges, and universities in planning to modify the quality of dental services.

## 2 Methods

### 2.1 Study Design

This applied study was done using a cross‐sectional descriptive‐analytical design. The questionnaires were distributed to the target population without randomization or blinding. All participants had equal and unrestricted access to the questionnaire, with no allocation or stratification applied. The questionnaires were completed anonymously to keep the participants' information confidential. The protocol for the present study was approved by the Research Ethics Committee of Kerman University of Medical Sciences with the Ethical Code: IR.KMU.REC.1400.279. Furthermore, informed consent was obtained from all individuals.

### 2.2 Participants

The contributors in this study were selected employing census (complete enumeration) sampling from professors, students, and nurses in different clinical departments (Pathology, Orthodontics, Pediatrics, Surgery, Endodontics, Restorative dentistry, Prosthetics, Periodontics, and Radiology) at the School of Dentistry of Kerman University of Medical Sciences during 2021–2022. To do so, a list of all professors and nurses working in the school was received from the recruitment office, as well as a list of dental students from the office of admission and school records. Inclusion in the analysis was contingent solely on completion of the questionnaire; respondents who returned incomplete questionnaires or failed to return them were excluded from the final analysis. This approach minimized selective data exclusion based on individual characteristics and ensured the completeness and adequacy of the dataset for statistical analysis.

### 2.3 Study Size

The sample consisted of 60 professors, 300 students, and 50 nurses, totaling 410 individuals, as recorded by the Kerman School of Dentistry. A census (complete enumeration) sampling method was used for the current study. Based on Krejcie and Morgan's sampling table, a minimum sample size of 199 is sufficient to achieve adequate statistical power to test the study hypotheses for a population of 410 [17]. Additionally, a power analysis conducted using G*Power version 3.1.9.7 (Heinrich Heine University Düsseldorf, Germany) indicated that, for an ANOVA fixed effects, omnibus, one‐way test—assuming a medium effect size ( *f* = 0.25), an alpha level of 0.05, and a power of 0.95 across three groups—a minimum of 252 participants would be required.

### 2.4 Variables and Measurements

The SERVQUAL survey was used to evaluate the quality of dental services provided in the School of Dentistry. The validity and reliability of the localized version of the instrument were confirmed by Gorji et al. [18]. The validity of the instrument was assessed and confirmed by subject‐matter experts, and its reliability was also established with Cronbach's alpha coefficient of 0.945 using the split‐half method [18]. Furthermore, Dopeykar et al. confirmed the content validity of the revised version of the instrument by surveying subject‐matter experts, including faculty members at the dental school and medical services management department (content validity index = 0.75 and content validity ratio = 0.72). The reliability of the instrument was also confirmed using the interitem consistency score and Cronbach's alpha coefficient (*α* = 0.93) [15].

The data in this study were prepared using a demographic information form and the SERVQUAL Survey. The demographic information list was used to register the type of medical departments/wards, gender, age, occupation, education, marital status, service records in the faculty, and employment status. Before collecting the data, the researcher attended different clinical departments and provided some instructions to the participants about the objectives and significance of the research project. Verbal informed consent was also obtained from all the participants. Afterward, the researcher distributed the demographic information form and the SERVQUAL survey to the participants and asked them to complete the items carefully, also answered any questions asked by the participants when completing the items. Finally, the collected data were codified and analyzed.

### 2.5 Statistical Analysis

Data analysis was performed with SPSS 27 (IBM Corp., Armonk, NY, USA) software using inferential and descriptive statistics. The quantitative variables were described as mean ± standard deviation, and the qualitative variables were assessed using frequency (percentage). To assess the assumption of normality, descriptive indices of skewness and kurtosis were calculated for each variable, and standardized values were examined. Normality was judged acceptable when standardized skewness and kurtosis fell within ± 2, following recommended guidelines [19]. The Chi‐square test, one‐way ANOVA, Tukey's post hoc test, and Linear regression analysis were also employed for data analysis. The significance level in all statistical procedures was considered at *p* < 0.05.

## 3 Results

**Figure 1:**
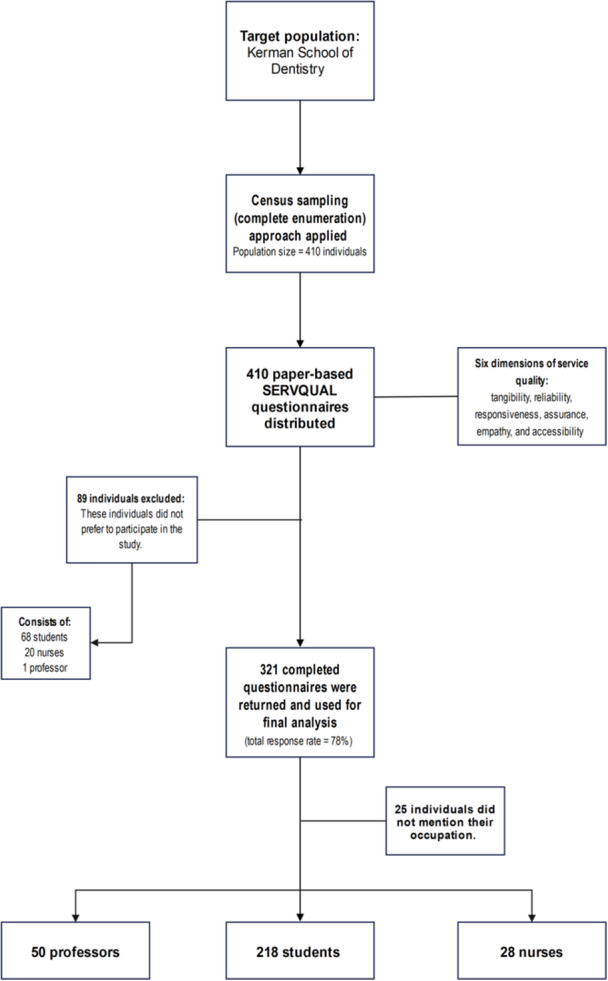
The study's flow diagram. SERVQUAL, service quality.

**Figure 2:**
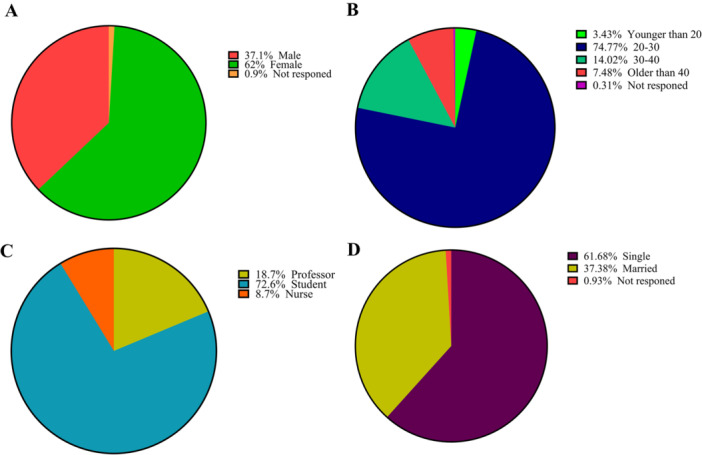
Pie charts of demographic information of participants. Sex (A), age (B), occupational status (C), and marital status (D). The data are shown as percentages.

Of a total of 410 questionnaires distributed among the participants, the data from 321 questionnaires were used for data analysis, while 89 incomplete questionnaires were excluded from the study (response rate: 78%). Approximately 62% of the participants were female, and 73% of the participants were students who were mostly admitted to the university in 2015 and 2016. Moreover, the largest number of participants in the study were aged 20–30 years, and the lowest number of participants were in the age group below 20 years. The data also showed that 98% of the students were completing their basic science courses, 73% of the professors in this study were general dentists, and 49% of the nurses had a diploma degree. Besides, 88% of the professors were married, and 78% of the students were single. The professors and nurses in this study were significantly older than the students (*p* = 0.001). However, there was no statistically significant difference between the three groups of participants in terms of gender (*p* = 0.63). In addition, the number of single individuals was significantly higher among students compared to the other two groups. (*p* = 0.001). Figures 1 and 2 present key aspects of the study. Figure 1 illustrates the study's flow diagram, while Figure 2 displays the demographic characteristics of the study population.

### 3.1 Participants' Perceptions of the Quality of Dental Services

As shown in Table 1, professors reported the highest mean scores in five dimensions—tangibility (17.28 ± 3.54), reliability (18.50 ± 3.69), responsiveness (19.18 ± 3.14), assurance (18.45 ± 3.03), and accessibility (12.28 ± 2.58)—suggesting a more favorable perception of service delivery among them. Nurses demonstrated relatively higher ratings in empathy (13.22 ± 3.06). In contrast, dental students consistently reported the lowest scores across all dimensions. The overall SERVQUAL score was highest among professors (96.38 ± 15.44), followed by nurses (93.21 ± 17.54; equivalent to 96.7% of the professors' score), while dental students reported the lowest mean score (85.45 ± 13.49; equivalent to 88.6% of the professors' score). The aggregate mean across all participants was 87.55 ± 14.88. At the population level, assurance (17.72 ± 2.91) and responsiveness (16.36 ± 3.68) emerged as the strongest dimensions, whereas accessibility (11.38 ± 2.52) and empathy (12.53 ± 2.84) were identified as relative weaknesses. Normality testing using skewness and kurtosis suggested that all dimensions followed a normal distribution (Table 2). Levene's test was performed to assess the homogeneity of variances and indicated that variances were homogeneous across all variables (Table 3). The one‐way ANOVA test shows that there is a significant statistical difference between at least two groups of nurses, students, and professors in the dimensions of tangibility, reliability, responsiveness, and accessibility (Table 4). Furthermore, the observed powers for the dimensions of empathy and assurance were 0.195 and 0.420, respectively, as reported in Table 4. Additionally, Tukey's post hoc test was used to accurately detect the difference between the groups (Table 5). Table 5 also shows the scores of the professors' and students' perceptions about the quality of dental services. As can be seen, there were significant differences in tangibility, reliability, responsiveness, and accessibility. However, no significant difference was observed between the two groups regarding their attitudes toward empathy and assurance. Besides, there was no statistically significant difference between the professors and nurses in terms of their perceptions of the dimensions of the quality of dental services. A significant difference was found between the students and nurses regarding their attitudes toward tangibility, responsiveness, and accessibility.

**Table 1:** Descriptive statistics for the dimensions of the quality of dental services.

**Dimensions**	Professors			Students			Nurses			All participants		
	*N*	Mean ± SD	Min, Max	*N*	Mean ± SD	Min, Max	*N*	Mean ± SD	Min, Max	*N*	Mean ± SD	Min, Max
Tangibility	49	17.285 ± 3.541	9, 23	216	14.222 ± 3.304	5, 21	27	16.259 ± 3.346	10, 22	317	14.713 ± 3.580	5, 23
Reliability	50	18.500 ± 3.693	8, 25	215	15.991 ± 3.082	7, 25	27	17.296 ± 3.279	8, 23	317	16.422 ± 3.324	7, 25
Responsiveness	49	19.183 ± 3.147	7, 25	216	15.629 ± 3.252	5, 23	27	18.333 ± 4.197	5, 25	317	16.362 ± 3.679	5, 25
Assurance	48	18.458 ± 3.038	10, 25	212	17.603 ± 2.632	5, 24	28	18.214 ± 4.094	5, 24	313	17.728 ± 2.907	5, 25
Empathy	50	12.380 ± 3.545	4, 19	212	12.523 ± 2.633	5, 18	27	13.222 ± 3.067	4, 18	313	12.536 ± 2.839	4, 19
Accessibility	49	12.285 ± 2.582	4, 19	212	11.113 ± 2.374	4, 19	28	12.214 ± 3.166	4, 18	314	11.382 ± 2.527	4, 19
Total	50	96.380 ± 15.441	42, 131	218	85.454 ± 13.493	33, 112	28	93.214 ± 17.542	36, 124	321	87.551 ± 14.886	33, 131

Abbreviations: Max, maximum; Min, minimum; SD, standard deviation.

**Table 2:** Descriptive statistics of skewness and kurtosis for normality testing of dimensions.

Dimensions	*N*	Mean	Skewness		Kurtosis	
			Statistic	Standard error	Statistic	Standard error
Tangibility	317	14.7129	–0.169	0.137	–0.262	0.273
Reliability	317	16.4227	–0.194	0.137	0.090	0.273
Responsiveness	317	16.3628	–0.327	0.137	0.080	0.273
Assurance	313	17.7284	–0.964	0.138	1.924	0.275
Empathy	313	12.5367	–0.592	0.138	0.181	0.275
Accessibility	314	11.3822	–0.025	0.138	1.054	0.274

Abbreviation: Std. Error, standard error.

**Table 3:** The status of homogeneity of variance for each dimension.

Dimensions	Levene statistic	df	*p*‐value
Tangibility	0.442	2	0.643
Reliability	0.415	2	0.661
Responsiveness	0.775	2	0.462
Assurance	2.986	2	0.052
Empathy	2.645	2	0.073
Accessibility	1.146	2	0.319

Abbreviation: df, degrees of freedom.

**Table 4:** Comparing the participants' perceptions of the quality of services using One‐Way ANOVA.

Dimensions		*N*	Sum of squares	df	Mean square	*F*	*p*‐value	Observed power
Tangibility	Between groups		427.824	2	213.912	19.077	< 0.001*	1
	Within groups		3240.519	289	11.213			
	Total	292	3668.342	291				
Reliability	Between groups		272.396	2	136.198	13.199	< 0.001*	0.997
	Within groups		2982.111	289	10.319			
	Total	292	3254.507	291				
Responsiveness	Between groups		607.115	2	303.557	27.349	< 0.001*	1
	Within groups		3207.717	289	11.099			
	Total	292	3814.832	291				
Assurance	Between groups		33.763	2	16.882	2.048	0.131	0.420
	Within groups		2349.348	285	8.243			
	Total	288	2383.111	287				
Empathy	Between groups		13.737	2	6.869	0.846	0.430	0.195
	Within groups		2323.329	286	8.124			
	Total	289	2337.066	288				
Accessibility	Between groups		74.342	2	37.171	5.972	0.003*	0.878
	Within groups		1779.997	286	6.224			
	Total	289	1854.339	288				

Abbreviations: df, degrees of freedom; F, *F*‐statistic.

* Significance at *p* < 0.05.

**Table 5:** Results of the Tukey's post‐hoc test for One‐Way ANOVA.

**Dimensions**	Professors‐Students		Professors‐Nurses		Students‐Nurses	
	Mean difference	*p*‐value	Mean difference	*p*‐value	Mean difference	*p*‐value
Tangibility	3.06	< 0.001*	1.02	0.408	–2.03	0.009*
Reliability	2.51	< 0.001*	1.20	0.261	–1.31	0.116
Responsiveness	3.55	< 0.001*	0.85	0.537	–2.70	< 0.001*
Assurance	0.85	0.152	0.24	0.932	–0.61	0.541
Empathy	–0.14	0.945	‐0.84	0.432	–0.69	0.454
Accessibility	1.17	0.009*	0.07	0.992	–1.10	0.074
Total	10.83	< 0.001*	3.41	0.548	–7.41	0.023*

* Significance at *p* < 0.05.

### 3.2 The Students' Perceptions of the Quality of Dental Services by Demographic Factors

As shown in Table 6, there was no significant relationship between dimensions of SERVQUAL and the students' demographic variables.

**Table 6:** The students' perceptions of the quality of dental services by demographic factors.

Dimensions	Age		Gender		Marital status		Employment		Academic level	
	*p*‐value^a^	*β*	*p*‐value	*β*	*p*‐value	*β*	*p*‐value	*β*	*p*‐value	*β*
Tangibility	0.955	–0.019	0.514	–0.360	0.938	0.028	0.498	0.383	0.728	–0.119
Reliability	0.721	–0.116	0.276	–0.583	0.921	–0.034	0.260	0.618	0.615	–0.164
Responsiveness	0.898	0.043	0.456	–0.405	0.833	0.075	0.435	0.435	0.665	–0.146
Assurance	0.844	0.069	0.989	0.008	0.758	0.115	0.972	0.020	0.745	–0.115
Empathy	0.956	0.018	0.520	–0.347	0.844	0.070	0.378	0.491	0.755	–0.105
Accessibility	0.514	–0.206	0.259	–0.581	0.635	–0.158	0.158	0.761	0.570	–0.179
Total	0.991	0.004	0.530	–0.342	0.797	0.093	0.499	0.378	0.646	–0.156

Abbreviation: *β,* regression coefficient.

^a^ Obtained using Linear regression.

### 3.3 The Professors' Perceptions of the Quality of Dental Services by Demographic Factors

The data in Table 7 indicate that older professors assigned significantly higher scores to reliability (*p* = 0.006). Moreover, older professors assigned significantly higher scores to the dimensions of quality of services (*p* = 0.032). However, no relationship was found between the remaining dimensions of SERVQUAL and the professors' demographic variables.

**Table 7:** The professors' perceptions of the quality of dental services by demographic factors.

Dimensions	Age		Gender		Marital status		Employment		Field of expertise	
	*p*‐value^a^	*β*	*p*‐value	*β*	*p*‐value	*β*	*p*‐value	*β*	*p*‐value	*β*	
Tangibility	0.175	0.338	0.301	–0.202	0.407	–0.208	0.896	0.050	0.825	0.088	
Reliability	0.006*	0.660	0.277	0.190	0.346	–0.212	0.321	0.338	0.659	–0.156	
Responsiveness	0.200	0.295	0.220	–0.223	0.107	–0.382	0.893	–0.047	0.678	0.152	
Assurance	0.142	0.392	0.582	0.114	0.937	0.021	0.218	0.504	0.274	–0.467	
Empathy	0.303	0.257	0.489	0.136	0.128	–0.392	0.290	0.407	0.706	–0.151	
Accessibility	0.389	0.227	0.565	0.119	0.386	–0.233	0.640	0.189	0.732	–0.145	
Total	0.032*	0.515	0.932	–0.015	0.207	–0.300	0.643	0.164	0.881	0.055	

Abbreviation: *β,* regression coefficient.

* Significance at *p* < 0.05.

^a^ Obtained using Linear regression.

### 3.4 The Nurses' Perceptions of the Quality of Dental Services by Demographic Factors

Table 8 shows that married nurses scored significantly higher than single nurses on accessibility (*p* = 0.021). In addition, female nurses scored significantly higher than male nurses on reliability (*p* = 0.016).

**Table 8:** The nurses' perceptions of the quality of dental services by demographic factors.

	Age		Gender			Marital status			Employment			Field of expertise	
Dimensions	*p*‐value^a^	*β*	*p*‐value	*β*		*p*‐value	*β*		*p*‐value	*β*		*p*‐value	*β*
Tangibility	0.851	0.080	0.929	–0.024		0.054	–0.515		0.525	0.178		0.994	0.003
Reliability	0.197	0.543	0.016*	0.677		0.677	–0.100		0.504	–0.180		0.389	0.304
Responsiveness	0.481	0.296	0.406	0.218		0.225	–0.305		0.868	0.045		0.716	–0.130
Assurance	0.184	0.569	0.319	0.262		0.597	–0.130		0.964	–0.012		0.951	0.022
Empathy	0.475	0.322	0.728	0.097		0.154	–0.387		0.924	–0.028		0.632	0.183
Accessibility	0.598	–0.216	0.926	–0.023		0.021*	–0.609		0.208	0.346		0.547	–0.211
Total	0.243	0.517	0.367	0.246		0.238	–0.308		0.900	–0.036		0.535	0.230

Abbreviation: *β*, regression coefficient.

* Significance at *p* < 0.05,

^a^ Obtained using Linear regression.

## 4 Discussion

In this study, 321 professors, students, and nurses participated, with 62% female and 73% students. Professors assigned the highest mean SERVQUAL score (96.38), students the lowest (85.45), and nurses scored 93.21. Among the six dimensions, service assurance received the highest mean score (18.46 for professors), while accessibility received the lowest (11.38 for students). Significant differences were observed between professors and students in tangibility, reliability, responsiveness, and accessibility, as well as between students and nurses in tangibility and responsiveness. No significant differences were found in empathy or assurance among the groups.

Our results showing a generally moderate overall SERVQUAL score with relatively high ratings for assurance and lower ratings for accessibility align with several recent dental and health‐service studies: Sharka et al. found assurance and tangibility strongly influenced patient return intention [20], and Wu et al. reported responsiveness and assurance as key predictors of patient satisfaction [21], another academic dental study likewise observed high interpersonal‐trust/competence scores but lower access‐related ratings. However, some regional and Iranian studies report different patterns, identifying empathy. or responsiveness as the weakest dimensions or showing no clear provider–trainee differences, which contrasts with our finding of lower student ratings for tangibility, reliability, responsiveness, and accessibility [22]. Such discrepancies likely reflect contextual factors (patient mix, measurement adaptations, clinic size, and appointment policies), underscoring the need for institution‐specific interpretation and justifying this study's local assessment.

The quality of service delivery has become increasingly important, especially in healthcare centers that deal with a great number of clients. Improving the quality of services can be considered a necessary strategy that helps any organization achieve better outcomes in the competitive market. SERVQUAL in the healthcare sector has a special place because this sector is responsible for preserving health and taking care of the lives of people in the community. The present study evaluated the quality of services provided to patients who visited the School of Dentistry of Kerman University of Medical Sciences from the perspective of professors, students, and nurses in 2021–2022. A total of 321 professors, students, and nurses participated in this study. The findings showed that the mean score for all dimensions of SERVQUAL from the perspective of professors, students, and nurses was 87.55 ± 14.88, confirming that the quality of healthcare services provided at the School of Dentistry in Kerman is slightly above average. Interestingly, accessibility obtained the lowest mean score among the six dimensions, suggesting that patients and providers perceive more barriers in this area compared to others. This finding aligns with previous studies emphasizing the need to improve access to oral health services [9, 20].

Parasuraman et al. introduced the SERVQUAL model in 1985. This model measures the level of SERVQUAL from the clients' perspective by analyzing the gap between the clients' expectations and perceptions [8].

A survey of the professors, students, and nurses in this study suggested that the overall score for the quality of services was 87.5 out of 140. The professors assigned the highest score (96.38) and the students assigned the lowest score (85.45) to the quality of service delivery. The highest score was assigned to service assurance (17.72), and the lowest score was assigned to service accessibility (11.38).

The findings from the present study also suggested significant differences in the perceptions of professors and students about tangibility, reliability, responsiveness, and access to dental services. Nevertheless, no significant difference was observed between these two groups in terms of the assurance and empathy of service providers. Furthermore, no significant difference was observed between the professors and nurses in terms of their perceptions of the quality of dental services. However, a significant difference was found between the contributors of the students and nurses about the tangibility and responsiveness dimensions. A study by Karydis et al. in Greece on patients' perceptions and expectations of the quality of dental care indicated that due to the different dimensions of healthcare services, the expectations of empathy and assurance of services were top priorities for patients [23]. However, a study by Gullu et al. in Turkey showed that patients had higher expectations about the empathy of service providers [24]. Akbar et al. showed that the dimensions of empathy and responsiveness in dental services in urban and rural areas were at an average level, indicating that empathy and responsiveness in dental services in hospitals and public health centers need to be improved [9]. Moreover, Tabibi et al. [25] and Güllü et al. [24] reported the highest level for responsiveness and the lowest level for reliability of healthcare services. Karydis et al. showed that responsiveness as a dimension of SERVQUAL obtained the lowest score [23]. Moreover, other studies conducted in Iran (e.g., Dopeykar et al. [15], Naqavi et al. [26], Bahadori et al. [27], and Bakhtiar et al. [28]) found that service assurance and empathy as components of SERVQUAL obtained the lowest scores. Studies conducted in different countries, including Indonesia [29], Bosnia and Herzegovina [30], Brazil [31], Jordan [32], and selected Asian countries [33] on the quality of dental services using the SERVQUAL model have shown a relationship between the quality of dental services and patient satisfaction. Sajadi et al. showed a significant relationship between perceived SERVQUAL and satisfaction, indicating that improving SERVQUAL can increase satisfaction and motivation to use dental services continuously [34]. In this study, an analysis of the perceptions of professors, students, and nurses confirmed service providers' unawareness of patients' conditions. Thus, such issues should be taken into account for future planning. A significant gap between the perceptions of patients and service providers about each dimension of SERVQUAL indicates that the dimension has received less attention and needs more focus and planning. Reducing the quality of different dimensions of service delivery has a synergistic effect and can reduce the quality in other dimensions.

The findings of this study showed a significant difference between the perception of professors and students in terms of the tangibility, reliability, responsiveness, and accessibility of dental services, and the students assigned a lower score to these dimensions. The data also indicated that students have a more negative assessment of the quality of service provision in the dental school compared to that of the professors. The lower scores assigned by students to several SERVQUAL dimensions may reflect their higher expectations and closer engagement in day‐to‐day clinical processes. Differences between students' and professors' perceptions could indicate varying levels of experience and familiarity with institutional constraints, rather than attitudinal differences. The finding that students rated SERVQUAL lower than professors may reflect their more idealistic expectations; as trainees, students often hold higher theoretical standards and concentrate on gaps between observed practice and idealized clinical guidelines, a perspective supported by prior studies showing that students generally adopt more critical views of SERVQUAL than professors due to limited practical experience and heightened awareness of best practices [20, 21]. Furthermore, within the service delivery triangle, students serve as a central component of care provision and possess direct knowledge of available equipment, patient communication, and patient satisfaction; consequently, any discrepancy between their perceptions and actual service delivery can intensify their negative appraisal and help explain the observed perceptual differences between students and professors.

The present study showed no significant difference in the perceptions of nurses and professors about the quality of services, indicating that professors and nurses have similar attitudes toward the quality of service delivery. Additionally, a significant difference was observed between the perceptions of students and nurses about tangibility and responsiveness; the students had a more negative assessment of these aspects of service delivery compared to the nurses, which can indicate a perception gap between students and nurses. Differences in SERVQUAL perceptions among professors, students, and nurses likely reflect variations in clinical experience, role expectations, and familiarity with service delivery. Students rated SERVQUAL lower than professors, possibly due to their limited practical exposure and idealized expectations of clinical care. Prior studies similarly show students tend to be more critical than faculty [9, 20, 21]. Institutional factors, like resource limitations and workload, may further widen these perceptual gaps. Enhancing communication and involving all stakeholders in quality improvement efforts could help bridge these differences.

An evaluation of the participants' demographic variables in the present study showed that there was no association between students' demographic variables and their perceptions of the quality of dental services. A study by John et al. in Malaysia showed no significant relationship between satisfaction and marital status [12]. Khaki et al. also reported no significant relationship between the marital status and the SERVQUAL's gap [35]. In addition, older professors assigned higher scores to the reliability of service delivery. Married nurses assigned higher scores to service accessibility. Female nurses assigned higher scores to the reliability of services, but no gender difference was observed in other dimensions of service delivery among all participants. The reason why professors and nurses had a more positive assessment of most areas of SERVQUAL compared to students can be attributed to the fact that professors and nurses have access to more facilities due to their job conditions, so they are more satisfied with services than students. In addition, professors and nurses at older ages have lower expectations of the quality of services provided, which can generally affect the differences in their perspectives. Although accessibility was included as one of the six SERVQUAL dimensions, it received relatively less emphasis in the discussion because the items measuring accessibility mainly focused on physical and organizational aspects (e.g., appointment availability, clinic location, and waiting times). These indicators may not have fully captured other important aspects of accessibility, such as affordability or digital access to dental services. Therefore, future studies should consider expanding the measurement of accessibility to include these dimensions for a more comprehensive evaluation.

This study has several important limitations that should be considered when interpreting the findings. Data were collected using self‐reported questionnaires and are therefore susceptible to response biases, including social desirability, self‐selection, and recall errors; moreover, 89 incomplete questionnaires (21.7%) were excluded, which may introduce selection bias and reduce sample representativeness. The accessibility dimension was measured primarily through participant perceptions and did not include objective indicators such as recorded waiting times, appointment capacity, or physical barriers; consequently, structural or organizational barriers may be underrepresented. There is also a risk that SERVQUAL items were interpreted differently by professors, students, and nurses, because variations in clinical experience, educational background, or familiarity with service‐quality concepts can influence responses. Although the SERVQUAL model is widely used in healthcare evaluation, some of its items may not fully capture the cultural and contextual characteristics of the Iranian healthcare system. Future multicenter studies are needed to validate and adapt the model. The cross‐sectional design prevents causal inference, as the results represent associations only. The single‐center setting (Kerman School of Dentistry) limits generalizability, and the absence of direct patient perspectives means the study does not provide a complete account of SERVQUAL from the users' point of view. Additionally, contextual factors such as local policies, resource availability, organizational culture, and faculty‐student ratios may have influenced participants' perceptions of SERVQUAL.

Given these limitations, observed perceptual differences may partly reflect differences in interpretation or reporting tendencies rather than true differences in service delivery; therefore, practical and organizational recommendations should be made cautiously. Future research should employ data triangulation (adding objective indicators such as waiting‐time records and appointment logs), longitudinal and qualitative designs to explore underlying causes of perceptual differences, pilot testing and cognitive interviews to ensure consistent item comprehension, minimal data collection from nonrespondents to assess selection bias, and multicenter studies alongside inclusion of patient perspectives to enhance validity and generalizability.

Based on the findings, university administrators and dental school leadership should prioritize improving accessibility by optimizing appointment systems, adjusting clinic hours, and removing physical access barriers. Concurrently, targeted training programs for faculty, students, and nurses should be developed to strengthen SERVQUAL competencies, particularly empathy and responsiveness, and these topics should be integrated into the curriculum to embed a patient‐centered culture in clinical education. Regular monitoring of SERVQUAL using validated instruments such as SERVQUAL is recommended to identify gaps and guide improvement, and structured communication channels among students, professors, and nurses should be established to reduce perceptual differences and promote collaborative service delivery. Combining these measures is expected to improve patient satisfaction, students' educational experience, and staff performance.

For future research, targeted experimental evaluations of educational, structural, and managerial interventions are advised; longitudinal and qualitative studies should explore the underlying causes of perceptual differences among students, faculty, and nurses; curriculum changes and service‐centered training should be assessed for effectiveness; and the impact of infrastructural reforms (appointment systems, equipment, physical access) on patient outcomes and educational processes should be measured. Given the observed student–professor perceptual gap and inconsistent findings in prior studies, forthcoming research should examine multiple dimensions of service delivery and produce evidence to inform institutional and national planning.

## 5 Conclusion

The present study indicated that professors, students, and nurses at the Kerman School of Dentistry had a moderately positive perception of dental SERVQUAL. Overall, service assurance received the highest score, while accessibility received the lowest. Professors rated tangibility, reliability, responsiveness, and accessibility significantly higher than students. No significant differences were observed between professors and nurses, whereas students rated tangibility and responsiveness lower than nurses.

## Author Contributions

**Fatemeh Sadat Sajadi:** conceptualization, Investigation, funding acquisition, writing – original draft, methodology, validation, writing – review and editing, project administration, data curation, supervision, resources, writing – review and editing. **Zahra Salari:** conceptualization, supervisions, investigations, methodology, validation. **Keyhaneh Ebrahimi:** conceptualization, investigations, writing – original draft, methodology. **Maryam Rad:** writing – review and editing, formal analysis, validation. **Hossein Akbari‐Hooye:** writing – review and editing, formal analysis, software. **Milad Mollaali:** conceptualization, writing – review and editing, validation, visualization, methodology, software, formal analysis, data curation. All authors have read and approved the final version of the manuscript.

## Conflicts of Interest

The authors declare no conflicts of interest.

## Transparency Statement

The lead author, Milad Mollaali affirms that this manuscript is an honest, accurate, and transparent account of the study being reported; that no important aspects of the study have been omitted; and that any discrepancies from the study as planned (and, if relevant, registered) have been explained. The lead author, Milad Mollaali had full access to all of the data in this study and takes complete responsibility for the integrity of the data and the accuracy of the data analysis.

## Data Availability

The data to support the findings in the study are available on request from the corresponding author.
